# In Situ Study of the Microstructural Evolution of Nickel-Based Alloy with High Proportional Twin Boundaries Obtained by High-Temperature Annealing

**DOI:** 10.3390/ma16072888

**Published:** 2023-04-05

**Authors:** Chao Zhang, Ming Sun, Ruhan Ya, Lulu Li, Jingyi Cui, Zhipeng Li, Wenhuai Tian

**Affiliations:** 1 School of Materials Science and Engineering, University of Science and Technology Beijing, Beijing 100083, China; 2 State Key Laboratory of Multiphase Complex Systems, Institute of Process Engineering, Chinese Academy of Sciences, Beijing 100190, China

**Keywords:** electron backscattered diffraction, twin boundary, grain rotation, cold deformation

## Abstract

In this paper, we report an in situ study regarding the microstructural evolution of a nickel-based alloy with high proportional twin boundaries by using electron backscatter diffraction techniques combined with the uniaxial tensile test. The study mainly focuses on the evolution of substructure, geometrically necessary dislocation, multiple types of grain boundaries (especially twin boundaries), and grain orientation. The results show that the Cr20Ni80 alloy can be obtained with up to 73% twin boundaries by annealing at 1100 °C for 30 min. During this deformation, dislocations preferentially accumulate near the twin boundary, and the strain also localizes at the twin boundary. With the increasing strain, dislocation interaction with grain boundaries leads to a decreasing trend of twin boundaries. However, when the strain is 0.024, the twin boundary is found to increase slightly. Meanwhile, the grain orientation gradually rotates to a stable direction and forms a Copper, S texture, and α-fiber <110>. Above all, during this deformation process, the alloy is deformed mainly by two deformation mechanisms: mechanical twinning and dislocation slip.

## 1. Introduction

Nickel-based alloy are widely used in demanding environments, such as aerospace, energy, and chemical industries, due to their excellent corrosion resistance and mechanical properties [1,2,3,4]. The material is usually cold drawn and cold rolled into wire and sheet. Moreover, it can obtain excellent mechanical properties through solid solution treatment combined with cold deformation [3,5]. Recently, many people have started to focus on research of cold deformation. The effect of cold deformation on nickel-based alloy is reported to refine the grain [6,7], optimize the ratio and distribution of precipitates [8], and the grain boundary (GB) distribution [3,9], which improves the mechanical properties of nickel-based alloy. From the above statements about nickel-based alloy, it is clear that the microstructure is inseparable from the excellent mechanical properties.

The Cr20Ni80 alloy is a stable austenitic alloy with a Cr replacement of Ni due to no phase change at different temperatures. It is a nickel-based alloy with excellent corrosion resistance and outstanding mechanical properties, and it is widely used in machinery, metallurgy, and industrial furnaces. As far as we know, there have been many studies reported on the cold working of the Cr20Ni80 alloy; for example, Zhou et al. [10,11] reported that Cr20Ni80 ultrafine alloy wire was annealed at different temperatures from 700 to 890 °C and then cold rolled to different diameters. The variety of GBs and grain orientations of the alloy wires with different diameters were investigated by XRD or TEM. The results showed that <111>, <311>, and <222> grains were coarsened while <200> and <220> disappeared when the annealing temperature was 890 °C, which contributed to the improvement of the mechanical properties of the Cr20Ni80 ultra-fine wires. In addition, the deformation mechanisms of the alloy are GB slippage and twinning. The above work is helpful for the preparation of ultra-fine alloy wires with excellent properties. However, the deformation behavior of Cr20Ni80 alloy is still not fully understood, especially for Cr20Ni80 alloys with a high proportion of twinned grain boundaries. Nickel-based alloy are low-stacking fault energy alloy with a face-centered cubic (fcc) crystal structure that can be annealed to obtain coherent twin boundaries (TBs) of up to 75% [12]. It is generally believed that TBs have lower surface energy and higher resistance to crack initiation and expansion compared to normal GBs. Moreover, the TBs can also hinder dislocation slip and improve material strength, similar to normal GBs during deformation. In engineering, the introduction of numerous TBs by grain boundary engineering (GBE) can effectively improve the service life of the alloy. However, some recent studies have found that TBs in polycrystalline nickel-based alloy are more likely to induce material failure [13]. Due to the complexity of the composition and microstructure of nickel-based alloy, the mechanism of the negative effect of TBs is not understood. This paper is devoted to determining the microstructure evolution of the Cr20Ni80 alloy with high proportional TBs.

GBs are interfaces separating grains of different crystallographic orientations that have an important effect on the plastic deformation of polycrystalline materials [14]. In addition, the main deformation mechanism of nickel-based alloy is the planar slip of dislocations [9]. During the deformation process, GBs may be a barrier to the glide of dislocations while dislocations preferentially accumulate near the GBs, which may lead to lattice rotation [15]. Meanwhile, there are various mechanisms of dislocation-GB interactions because of the multiplicity of GB types, which leads to different plastic deformation characteristics [16]. Therefore, a detailed analysis of GB evolution and lattice orientation rotation is essential to understanding the deformation behavior of the Cr20Ni80 alloy with high proportional TBs.

Electron backscatter diffraction (EBSD) is important in analyzing the mechanical properties and characterizing the microstructure of crystalline materials [17,18,19]. Furthermore, uniaxial stretching combined with EBSD characterization can allow observation of the microstructure and deformation characteristics evolution pattern with time. In recent years, the in situ EBSD technique has been widely used to study the microstructural evolution of crystalline materials [20,21]. This technique can provide some important information on the orientation and microstructural evolution under different strains in the same area, which contributes to our better understanding of the evolution of the microstructure and the mechanism of the GB with a dislocation reaction (especially TBs) for Cr20Ni80 alloy with high proportional TBs.

In this study, the evolution of GBs, substructure, and orientation of the Cr20Ni80 alloy during deformation is objectively and comprehensively analyzed and studied by combining high-temperature annealing (GBE) and in situ EBSD techniques so that the microstructural evolution and deformation behavior of the alloy with a high proportion of TBs can be better understood. Additionally, this study provides an experimental basis for simulation studies and contributes to the development of cold processing.

## 2. Experimental Procedure

### 2.1. Raw Materials

**Figure 1:**
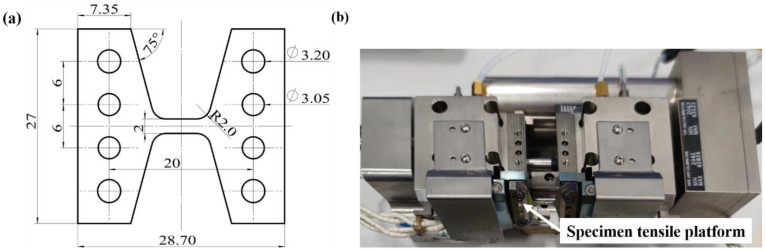
(**a**) Schematic diagram (in mm) of the in situ tensile test specimen. (**b**) In situ EBSD tensile system.

The material investigated in this paper is a commercial Cr20Ni80 alloy sheet with a thickness of 1 mm, which is usually prepared by the cold rolling technique. The alloy’s chemical composition (in wt.%) is 20.15 Cr, 0.27 Mn, 0.59 Fe, 0.29 Al, 1.17 Si, 0.032 C, 0.0032 S, 0.01 P, and Ni balance. The alloy is kept at 1100 °C for 30 min and then air-cooled (annealed treatment), subsequently machined by an electric discharge machine into a 10 mm (L) × 2 mm (W) in situ tensile sample, as shown in Figure 1a. These two directions— the rolling direction of the alloy sheet and the length direction of the in situ tensile specimen—are the same. To obtain a better and no-defect surface, the material is subjected to conventional mechanical grinding and electrolytic polishing. The electrolytic polishing parameters are −20 °C, 20 V, and the electrolyte consists of 10% perchloric acid and 90% alcohol solution.

### 2.2. In Situ Tensile Test

In situ tensile testing is performed at room temperature by an automated tensile system, which consisted of a tensile table mounted to an Ultra-55 Zeiss field emission scanning electron microscope (SEM). The EBSD tensioning table with a maximum load of 2000 N is shown in Figure 1b. During the tensile process, the specimen is tilted at 70°, the strain is measured by a sensor, and the deformation is performed at a rate of 0.1 mm/min. At each strain, the displacement is maintained for about 30 min to collect the EBSD data in the specimen of the same areas. Meanwhile, the initial resolution of the specimen is above 95%. The specimen coordinate system usually includes transverse direction (TD), rolling direction (RD), and normal direction (ND). TD is the direction perpendicular to ND and RD. RD is parallel to the tension loading direction of the specimen (or the rolling direction). ND represents the normal direction of the observed surface of the specimen. In situ EBSD data is analyzed in detail by HKL Channel5 software.

## 3. Results and Discussion

### 3.1. Microstructure and Mechanical Properties

**Figure 2:**
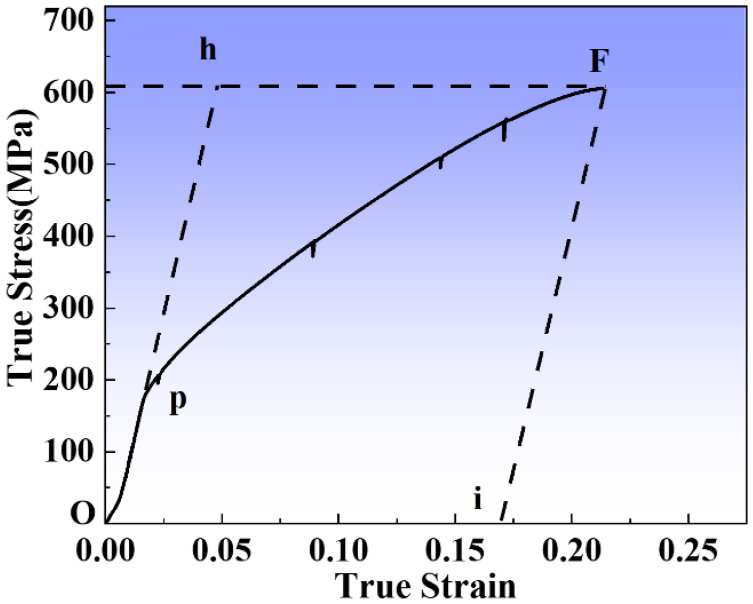
In situ tensile true stress–strain curve. (The OP of the curve corresponds to the elastic deformation and the PF corresponds to the elastoplastic deformation).

The true stress–strain curve of the specimen in the in situ EBSD test is shown in Figure 2. The OP of the curve corresponds to the elastic deformation and the PF corresponds to the elastoplastic deformation. To collect EBSD data, the strain needs to reach a preset value and keep a constant displacement for about 30 min. Some load reduction is observed in the PF stage of the true stress–strain curve during in situ EBSD testing, which is mainly attributed to stress relaxation. In addition, this phenomenon has been reported in several publications [20,22]. The tensile and yield strength of the Cr20Ni80 alloy with high-temperature annealing (GBE) treatment is 609 MPa and 193 MPa, respectively, while the maximum true strain is 0.215 by a discontinuous tensile, indicating that the specimen has excellent mechanical properties.

**Figure 3:**
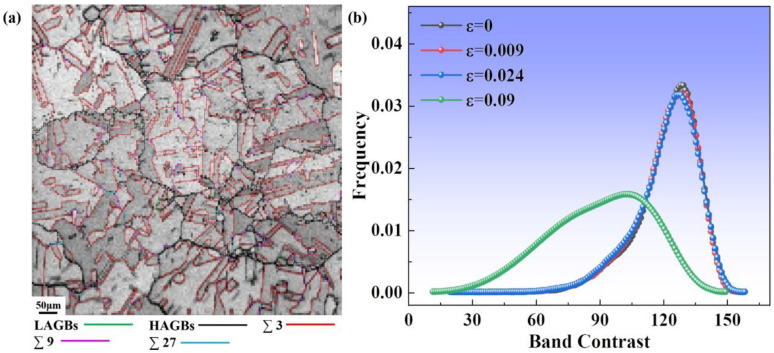
(**a**) The EBSD band contrast (BC) map of the original specimen in the annealed state. (**b**) Distribution of EBSD band contrast (BC) value under different strains.

The band contrast (BC) value is qualitatively correlated with the defect density in the signal excitation region, so the BC map can be used to qualitatively illustrate the magnitude of the defect density. The BC map is a two-dimensional grayscale map based on the reconstruction of the Kikuchi diffraction pattern by EBSD, which is similar to the SEM and secondary electron image. The BC map of the original microstructure of the specimen is shown in Figure 3, and the specific GBs can be fully presented in all grains. The BC map shows the quality of the Kikuchi pattern. As the grayscale changes from light to dark, this indicates the increase in the lattice distortion of the alloy specimen. It can be seen that the annealed specimen experienced recovery recrystallization and significant grain growth. The original microstructure of this specimen is mainly composed of coarse equiaxed grains and GBs mostly consisting of high-angle grain boundaries (HAGBs, >15°). In addition, the TBs are up to 73%. Figure 3b shows the distribution of BC values for different strains of the specimen. In the elastic phase, the peak decreases slightly; however, under large strains, the peak shifts to the left, and the grayscale becomes much lower. This indicates that the alloy exhibits numerous lattice distortions, defects, and plastic deformation [23].

### 3.2. Grain Orientation Distribution

**Figure 4:**
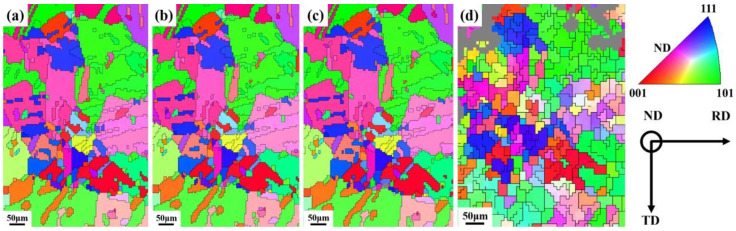
The EBSD Inverse Pole Figure (IPF) maps the same region of the specimen at different true strains: (**a**) 0, (**b**) 0.009, (**c**) 0.024, (**d**) 0.09. (RD: rolling direction, ND: normal direction, and TD: transverse direction; the IPF maps are plotted in the ND direction of the specimen).

The EBSD Inverse Pole Figure (IPF) maps are plotted in the ND direction of the specimen. Figure 4 represents the evolution of the orientation of the specimen in the same region under different strains. In the in situ EBSD study, the grain orientation rotation can be tracked by different colors, with red for [001], blue for [111], and green for [101] directions, respectively. Under large strain, the specimen undergoes significant lattice distortion due to the accumulation of a large number of lattice defects, leading to the destruction of the quality of the Kikuchi diffraction pattern [23], which has been discussed above. The gray area is the zero pixels point in the maps, which can be considered as lattice distortions occurring within the specimen, especially around the GBs, resulting from the high-stress concentration under cold deformation [24]. As shown in Figure 4a–d, the gray area that cannot be indexed gradually increases with increasing strain. The grain orientation is randomly distributed in the annealed condition, and no significant preferential orientation is observed. Meanwhile, with the increase in strain, the grains elongate along the RD direction. Furthermore, the grain is separated into numerous areas under large strain, which leads to the formation of sub-grains or substructures, as in Figure 4d.

### 3.3. Grain Boundaries Evolution

GBs are the interfaces separating grains with different grain orientations, which have important effects on the plastic deformation mechanism and mechanical properties of metallic materials [14]. Based on the misorientation angles, it can be roughly divided into high-angle grain boundaries (HAGBs) and low-angle grain boundaries (LAGBs). The binding energy between atoms in GBs with different misorientations varies greatly, which impacts atomic diffusion and nucleation. This means that the evolution of different types of GBs will have a great effect on the mechanical properties of the sample. In this study, the GBs with a misorientation angle between 2° and 15° are defined as LAGBs, while GBs with a misorientation angle larger than 15° are defined as HAGBs. In the HAGBs, the misorientation of 60 ± 5° along the <111> direction is defined as the twin boundary (TB).

**Figure 5:**
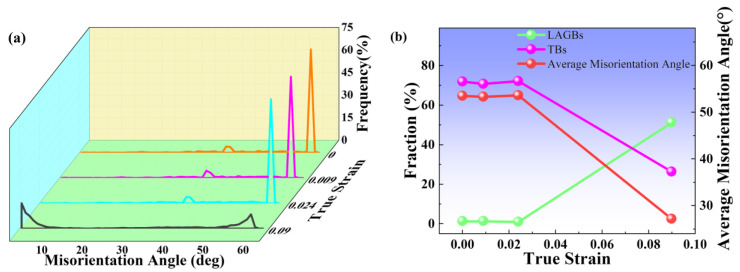
(**a**) Misorientation angle distribution of Cr20Ni80 alloy at various true strains. (**b**) The evolution of various grain boundaries at different true strains.

Figure 5 shows the evolution of different types of GBs in the in situ tests. Additionally, Figure 5a presents the distribution of misorientation angles between 2° and 62° at different strains. The GBs are mainly HAGBs (especially TBs), as can be seen. It is widely recognized that coherent TBs have lower interfacial surface energy and higher resistance to crack initiation and extension, which have important effects on the physical properties of the alloy. In the elastic deformation phase, there are two peaks in the misorientation angle distribution, which do not vary significantly at misorientation angles of 38° and 60°, respectively. However, when the strain reaches 0.09, the peak at a misorientation angle of 38° disappears, and the peak at a misorientation of 60° decreases significantly, while a peak appears at a misorientation of approximately 2°. This indicates that many substructures may be generated under large deformation.

Figure 5b shows the relationship between the true strain and the fraction of LAGBs and TB, as well as the average misorientation angle. In the low-stress state, the GB type is mainly dominated by HAGBs, especially TBs. Also, the proportion of LAGBs does not significantly vary, while TBs show a slight increase, and the average misorientation angle gradually increases. This indicates that deformation twins may form when the strain is 0.024. It is well known that fcc crystal nucleation during homogeneous deformation is closely related to the activation energy of 1/6<112> Shockley partial dislocations. Critical stress achieves γ/b to generate twinning (b is the modulus of the Shockley partial dislocation, γ is the energy of the stacking fault). Under the applied stress, the twin dislocations form a coherent interface parallel to the {111} close-packed plane by expanding and extending; in addition, new layers along the normal direction of the twin plane are formed by mechanisms to combine the twin planes to form a macroscopic twin [25,26,27]. The process of twin dislocation formation can be expressed as [28]:

(1) $$1/2\left[110\right]=1/3\left[111\right]+1/6[11\bar{2}]$$

Under large stresses, TBs are destroyed, the grains are refined, and the proportion of TBs is dramatically reduced, while LAGBs are significantly increased. TBs can absorb movable dislocations [29], and some of the moving dislocations along TBs can result in the migration of TBs [30,31]. Furthermore, LAGBs are generated owing to sub-grain rotation within the deformed grains to maintain the continuity of the sample matrix [9]. During plastic deformation, the TBs may migrate or annihilate by dislocation interactions. The evolution from HAGBs to LAGBs may develop extensive substructures.

### 3.4. Substructure Evolution

Kernel average misorientation (KAM) is commonly used to characterize the specimen’s local strain distribution and dislocation density [32]. The KAM value can qualitatively evaluate the sample deformation. This means that it can be closely related to coordinated deformation of the grains, dislocation slip, and lattice rotation. In this study, we constructed KAM maps of the specimen at various strains with a maximum misorientation of 5° between the target pixel and its neighboring pixel points.

**Figure 6:**
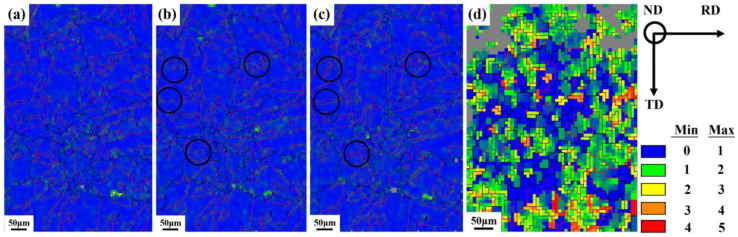
Kernel average misorientation (KAM) map of the same region of the specimen at various true strains: (**a**) 0, (**b**) 0.009, (**c**) 0.024, (**d**) 0.09 (RD: rolling direction, ND: normal direction, and TD: transverse direction).

Figure 6 shows the KAM maps of different strain samples, where HAGBs, TBs, and LAGBs are marked in black, red, and purple, respectively. The KAM values can be identified with different colors to observe the sample stress distribution. It can be seen that when the sample is loaded, the stress concentration occurs first at the TB, as shown in Figure 6b,c. As the strain increases, the dislocations tend to drive preferentially at the TBs, causing significant strain localization. It can be observed from Figure 6c that specially oriented {111} <112> twins may be present in the sample, which is a typical deformation behavior of fcc materials. In addition, when the strain reaches 0.09, the red and yellow regions increase significantly, while the proportion of TB decreases significantly, LAGB increases, and grain refinement occurs in Figure 6d. The surface quality of the sample is reduced, and there are many unindexed areas due to the large amount of deformation that occurs in the sample. Under large strains, the local average misorientation angle increases; meanwhile, the number of substructures increases.

As is well known, the dislocation density is closely linked to the strain, and the dislocation density is positively correlated with the average local misorientation. To quantitatively study the evolution of dislocation density during deformation, the average local misorientation measures are used to investigate the geometrically necessary dislocation (GND) variation during deformation, and the EBSD test data is used for the calculation. The GND density can be expressed as follows [22]:

(2) $$\rho^{\mathrm{GND}}=2\theta /\mu b$$

where *ρ*^GND^ is the GND density, b is Burger’s vector, *μ* is the unit length of the point, and *θ* is the average local misorientation angle.

**Figure 7:**
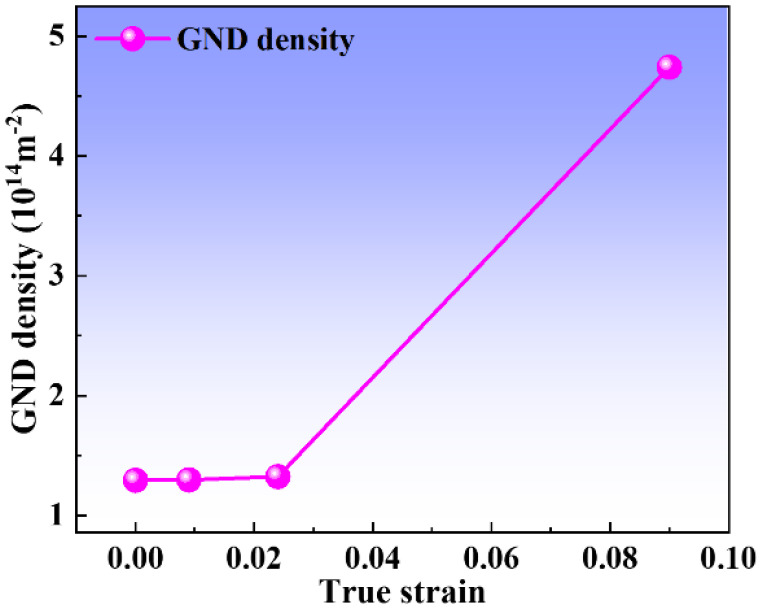
The evolution of geometrically necessary dislocation (GND) densities at different true strains.

The relationship between strain and GND density is shown in Figure 7. It indicates that the GND density gradually increases with the increase in strain. During polycrystalline deformation, GNBs are formed between regions with different strain patterns to accommodate the accompanying differences in lattice rotation [16]. Flow stress has an important effect on GNBs. As the strain increases, the dislocation source is continuously and substantially activated, and the dislocations proliferate. At low and moderate strains, the GND density increases slightly, while dislocations multiply rapidly when the strain exceeds 0.09. As a result, the dislocation proliferates explosively while most of the grains are refined under large strain, which can be linked to two strengthening mechanisms (dislocation strengthening and GB strengthening) [22], causing a strong work-hardening effect.

### 3.5. Grain Evolution during Deformation

A Recrystallized Fraction (DefRex) Map is usually used to characterize each grain’s microstructure changes during the deformation process. In this paper, according to the misorientation angles of each adjacent tiny region within the grains, the grains are divided into recrystallized (<1°), substructured (1°–7.5°), and deformed (>7.5°) grains, which are marked in blue, yellow, and red, respectively, in the DefRex Map.

**Figure 8:**
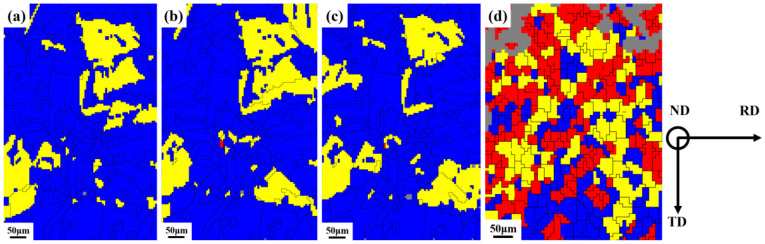
Distribution of recrystallized (blue), substructured (yellow), and deformed (red) (**a**) 0, (**b**) 0.009, (**c**) 0.024, (**d**) 0.09 (RD: rolling direction, ND: normal direction, and TD: transverse direction).

Figure 8 shows the grain evolution in the microstructure under different strains. At low and moderate strains, the samples are mainly dominated by recrystallized structure, indicating that recrystallization behavior occurred in the alloy during the high-temperature annealing process. During cold deformation, the concentration of vacancies in the matrix increases, providing a driving force for the diffusion of solid solution atoms [8]. When the strain reaches 0.09, the deformed grains suddenly increase, and more substructured grains appear around the deformed grains, as shown in Figure 8d.

**Figure 9:**
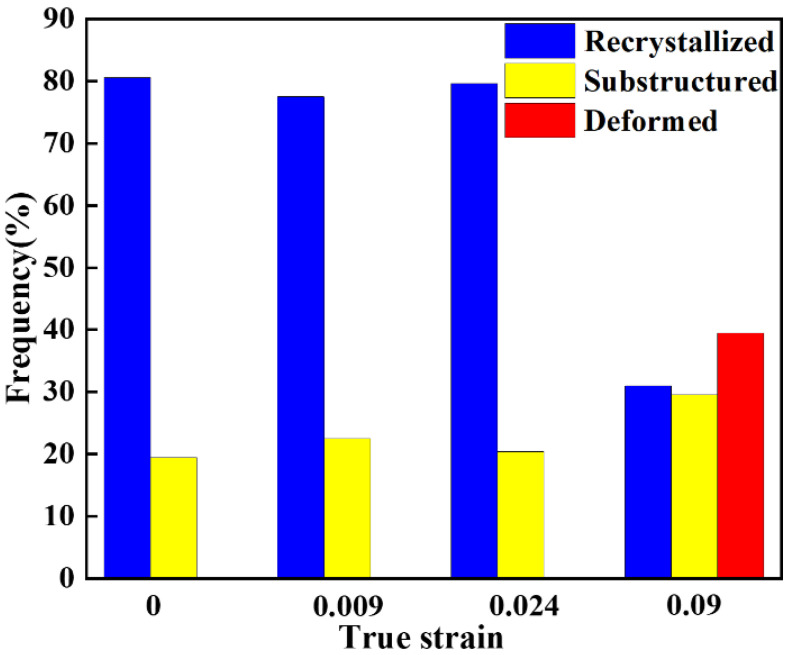
The evolution of grains at different true strains.

To further analyze the evolution of the microstructure during deformation, the distribution of different grains under different strains is statistically analyzed, as shown in Figure 9. It shows the relationship between strain and recrystallization, deformation, and substructure. As the strain increases, the recrystallized grains gradually decrease, while the substructured grains show an increasing trend, which is partially consistent with the GB evolution discussed above. Also, the deformed grains increase significantly when the strain reaches 0.09. Figure 8b,c and Figure 9 show that dislocations rearrange local regions during the interaction with dislocations and GBs, at low and moderate strains. The proportional recrystallization and the substructure are not changed significantly because the dislocation density increased slightly at different strains, ranging from low to medium. As the strain increases, the lattice in the grain rotates, and the proportion of the substructure increases. Moreover, when the lattice rotation is hindered, a lot of grains will deform under large stress. The effect of true strain on grain structure is strongly due to the increase in deformation stored energy with increasing true strain.

### 3.6. Schmid Factor

It is well known that the level of shear stress exerted on the slip surface and direction determines the plastic deformation of the crystalline material [33]. Nickel-based alloy are fcc metals with 12 slip systems, and {111} <110> is their main slip system. The single crystal is deformed by stretching based on the critical shear stress law, which can be expressed as [34]:

(3) $$\sigma_{0.2}=\tau_{c}/(\cos(\varphi )\cdot \cos(\lambda ))$$

where *σ*_0.2_ is yield strength, τ_c_ is critical shear stress (determined by the essential properties of the sheet), *φ* and *λ* are the angles formed by the stretching direction and the normal direction of the slip plane {111} and the slip direction <110>, respectively, and (cos(*φ*)cos(*λ*)) is the Schmid Factor.

**Figure 10:**
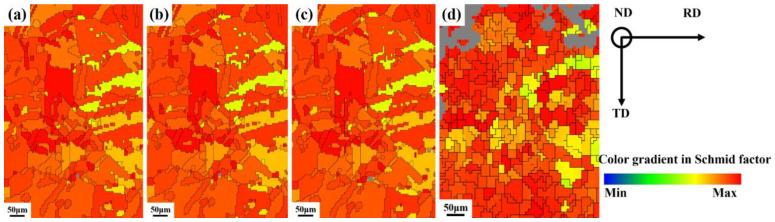
Schmid factor map of the same region of the specimen at various true strains: (**a**) 0, (**b**) 0.009, (**c**) 0.024, (**d**) 0.09 (RD: rolling direction, ND: normal direction, and TD: transverse direction).

The Schmid Factor (SF) represents the probability that the crystalline material will initiate a slip system in a certain direction (how difficult it is to deform). The higher the SF value, the higher the probability of the slip system activation. As shown in Figure 10, the SF value is based on the statistics of the Schmid factor for all pixels in the scanned area of the EBSD, the maximum value among all slip systems, which shows the SF determined at different strains of deformation.

**Figure 11:**
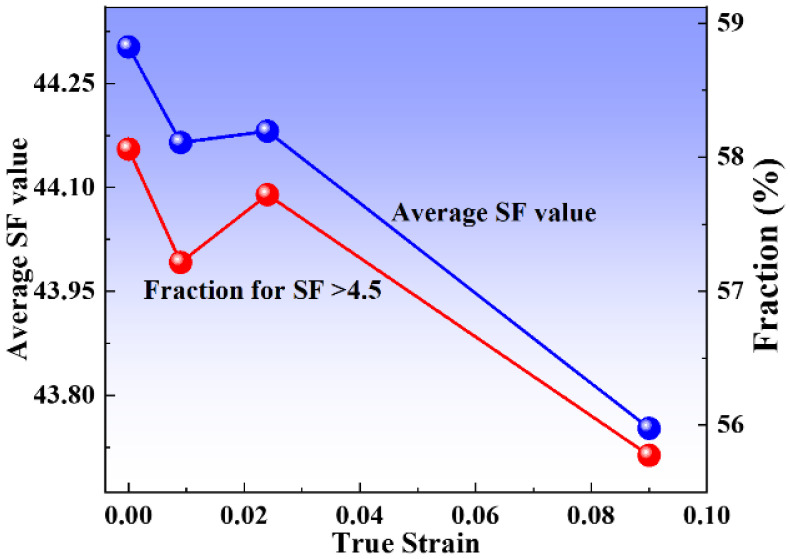
The evolution of the Schmid Factor (SF) values at various true strains.

The grain with a higher SF value means the grain is soft orientation, comparing the value of SF in different states. It can be seen that the SF value does not change significantly at low and medium strains, while at large strains, the yellow regions suddenly increase and some green regions appear, indicating that the SF value gradually decreases with increasing strain. We performed statistical calculations on SF values to further accurately analyze the relationship between SF values and different strains. Figure 11 shows that the SF value tends to decrease gradually with increasing strain but takes a sudden increase when the strain is 0.024. This may be attributed to the mechanical twinning of the sample during the deformation process.

In general, there are 3 to 5 slip systems operating simultaneously in each region within the grain during the deformation process [35]. With increasing stress, dislocations preferentially accumulate near twin boundaries, and different slide systems combine to slip, which causes lattice rotation. There are some differences in the slip systems that operate in different strain patterns. Under large stresses, different slip systems operate in combination, and adjacent grains interact with each other with uneven stresses, destroying grains. The SF values gradually stabilize and lower levels in the sample deformation. In polycrystalline deformation, the lattice needs to adjust the orientation to change the SF value to induce and activate the slip system, which causes the dislocation to start slipping and coordinates the deformation between grains.

### 3.7. Texture Evolution

**Figure 12:**
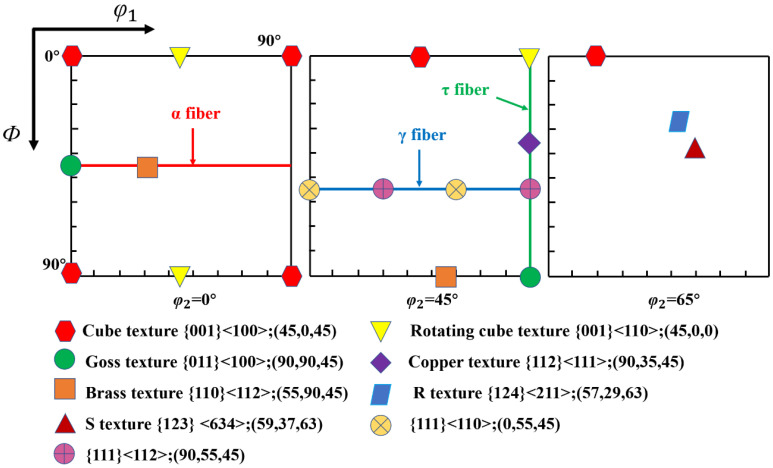
The distribution of ideal texture components of the nickel-based alloy; orientation distribution function (ODF) maps at *φ*_2_ = 0°, 45°, and 65°.

In crystallography, the orientation of most grains is usually concentrated in a direction—this phenomenon is called texture. The texture is closely related to the stress state of the sample [36]. In polycrystalline materials, the grains rotate to coordinate deformation between grains and ensure uniform sample deformation. The evolution of the texture of the Cr20Ni80 alloy during uniaxial tensile deformation is represented in the form of a cross-sectional map of the orientation distribution function (ODF) in the Euler angle space (*φ*_2_ = 0°, 45° and 65°). For clarity, the ideal basic texture components of the nickel-based alloy are shown in Figure 12.

**Figure 13:**
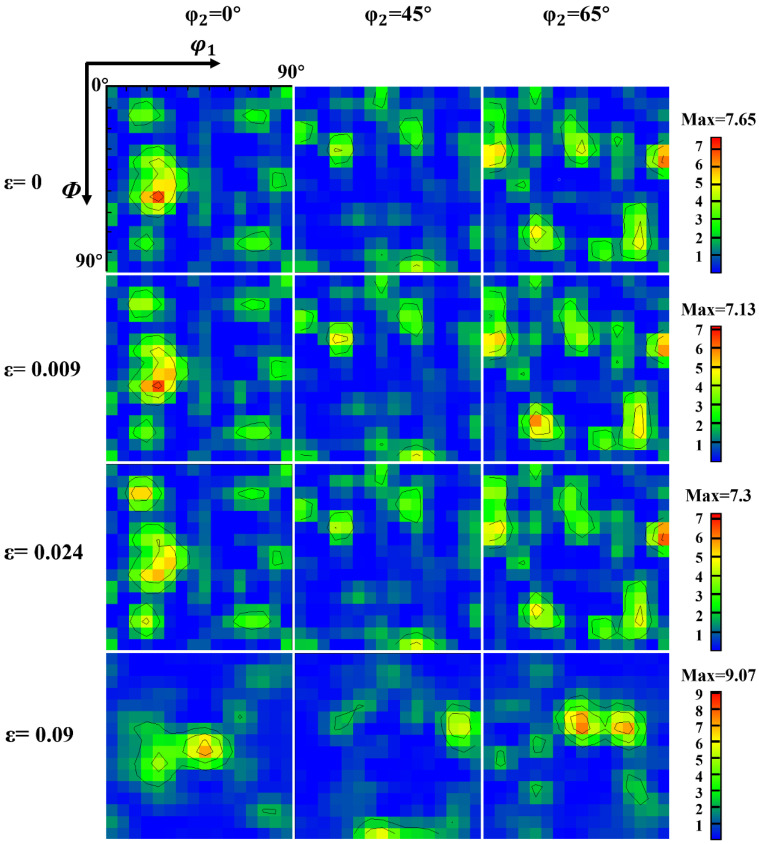
The evolution of the Cr20Ni80 alloy texture for different stress states.

Figure 13 shows the evolution of the sample texture for different stress states. The annealed state material texture is mainly composed of Brass {110} <112>, cube texture {001} <100>, R {124} <211> and slight Copper {112} <111>, respectively, by comparing with Figure 11. Normally, according to the dominant deformation mechanism, there are two different types of cold-rolled texture in fcc alloy or metals. This is determined by the cross-slip of screw-type dislocations. In addition, Cross-slip is influenced by various factors such as deformation conditions (temperature and strain rates [37]) as well as material properties (second phase particle, low stacking fault energy). However, the sample is an fcc crystal structure with low stacking fault energy alloy, which may hinder the cross-slip of screw-type dislocations [38]. Therefore, when the strain increases to 0.024, mechanical twinning of the polycrystalline material occurs, and the intensity of the Brass texture {110} <112> is reduced. With the increase in strain, cold deformation texture is gradually formed, such as α-fiber (RD//<110>), R {124} <211>, S {123} <634>, and Copper {112} <111>.

The Copper and S orientations have low SF values, whereas the Brass and Goss orientations have high SF values. Consequently, it is difficult to deform the grains in the Copper and S directions during cold deformation, which tends to cause uneven stresses that promote shear band formation and increase the local strain in the specimen [39]. Generally, Copper and S orientation grains are found in the large strain regions, while Goss and brass orientation grains are localized around the low strain regions. During the deformation process, dislocation multiplication and interaction with dislocations and GBs lead to lattice rotation with increasing strain, resulting in a gradual decrease in the intensity of the Brass texture and an increase in the intensity of the Copper and S texture. Meanwhile, the slip system {111} <110> is activated so that dislocations start to slip along the {111} slip plane, and the grain orientation gradually rotates to a stable direction forming the α-fiber <110>. Therefore, for the Cr20Ni80 alloy containing 73% TBs, the grain orientation gradually rotates toward the low Schmid Factor (SF) direction with increasing strain. In other words, the grains in the material gradually rotate from cubic {001} <100> and Brass {110} <112> direction to Copper {112} <111>, S {123} <634>, and α-fiber (RD//<110>) direction during cold deformation.

## 4. Conclusions

In this paper, the microstructural evolution of a nickel-based alloy with a high proportion of twin boundaries was tracked by in situ EBSD and a uniaxial tensile test. Additionally, the sample was treated by high-temperature annealing (grain boundary engineering) to obtain up to 73% twin boundaries. Grain rotation, grain boundary evolution, and substructure transformation were studied and analyzed in detail. The microstructural evolution of the alloy was carefully studied during deformation, and the main conclusions are as follows:

1. During the deformation of the Cr20Ni80 alloy, the proportion of low-angle grain boundaries gradually increases, the twin boundaries tend to reduce, and the substructure gradually increases. Meanwhile, mechanical twinning is found to occur at low strain. The alloy is deformed mainly by two deformation mechanisms: dislocation slip and mechanical twinning.
2. Dislocations preferentially accumulate near the twin boundary, and the strain localizes at the twin boundary with increasing strain. Meanwhile, at low strains, the geometrically necessary dislocation increases slightly with increasing strain. However, the geometrically necessary dislocation proliferates explosively under large strains, the different slip systems operate in combination, and adjacent grains interact with each other with uneven stresses, which destroys many grains. This above phenomenon can be linked to two strengthening mechanisms (dislocation strengthening and grain boundary strengthening), causing a strong work-hardening effect.
3. The Copper and S orientations have low Schmid Factor values, whereas the Brass and Goss orientations have high SF values. In addition, dislocation proliferates, and interaction with dislocations and grain boundaries leads to lattice rotation, resulting in a gradual decrease in the intensity of the Brass texture and an increase in the intensity of the Copper and S texture during deformation. Meanwhile, with strain increasing, the slip system {111} <110> is activated so that dislocations start to slip along the {111} slip plane, and the grain orientation gradually rotates to a stable direction, forming the α-fiber <110>. Therefore, the grains gradually rotate toward lower Schmid Factor values during plastic deformation, eventually forming the cold deformation texture such as α-fiber (RD//<110>), R {124} <211>, S {123} <634>, and Copper {112} <111>.

## Data Availability

Not applicable.
